# Aphid Resistance: An Overlooked Ecological Dimension of Nonstructural Carbohydrates in Cereals

**DOI:** 10.3389/fpls.2020.00937

**Published:** 2020-06-25

**Authors:** Victor O. Sadras, Elias Fereres, Lucas Borrás, Elisa Garzo, Aranzazu Moreno, Jose Luis Araus, Alberto Fereres

**Affiliations:** ^1^ South Australian Research and Development Institute, The University of Adelaide, Adelaide, SA, Australia; ^2^ IAS-CSIC, Cordoba, Spain; ^3^ ETSIAM, University of Cordoba, Cordoba, Spain; ^4^ Facultad de Ciencias Agrarias, Universidad Nacional de Rosario, Campo Experimental Villarino, , Buenos Aires, Argentina; ^5^ Instituto de Ciencias Agrarias, CSIC, Madrid, Spain; ^6^ Plant Physiology Section, Faculty of Biology, University of Barcelona, Barcelona, Spain; ^7^ AGROTECNIO Center, Lleida, Spain

**Keywords:** osmotic potential (OP), trade-off, grain yield (GY), cereal, labile carbohydrates

## Abstract

Nonstructural carbohydrates in cereals have been widely investigated from physiological, genetic, and breeding perspectives. Nonstructural carbohydrates may contribute to grain filling, but correlations with yield are inconsistent and sometimes negative. Here we ask if there are hidden functions of nonstructural carbohydrates, advance an ecological dimension to this question, and speculate that high concentration of nonstructural carbohydrates may challenge the osmotic homeostasis of aphids, thus providing a working hypothesis that connects nonstructural carbohydrates with aphid resistance in cereals. In the light of this proposition, the amount and concentration of nonstructural carbohydrates should be regarded as functionally different traits, with amount relevant to the carbon economy of the crop and concentration playing an osmotic role. We conclude with suggestions for experiments to test our hypothesis.

## Nonstructural Carbohydrates Play Many Roles in Plants, but Associations With Yield Are Ambiguous and Sometimes Negative In Cereals

Plants accumulate nonstructural carbohydrates during specific developmental stages (Hall et al., 1989; Dreccer et al., 2009; Slewinski, 2012) and when stresses such as nitrogen deficit, drought, and low temperature decouple growth and photosynthesis (Barlow et al., 1976; Pontis, 1989; Sadras et al., 1993; Muller et al., 2011; Körner, 2015). Scheduling harvest in relatively cool and dry periods and irrigation management to generate mild water stress before harvest illustrate agronomic practices seeking to shift carbon allocation from structural growth to sucrose accumulation in sugar cane (Inman-Bamber, 2004). In cereals, nonstructural carbohydrates conspicuously accumulate in shoots of nitrogen-deficient plants (van Herwaarden et al., 1998; Dreccer et al., 2013; Hoogmoed and Sadras, 2016; Ovenden et al., 2017; Sadras et al., 2017) and in the shoot and root of plants exposed to low temperature (Pontis, 1989; Tognetti et al., 1990; Equiza et al., 1997). Nitrogen deficit and low temperature also favor the storage of carbohydrates in woody perennials (Korner, 2003; Kobe et al., 2010). However, insoluble carbohydrates such as starch typically stored in woody perennials are osmotically inactive (Korner, 2003), whereas fructans and sucrose have significant osmotic effects in cereals (Pontis, 1989; Storlie et al., 1993).

Ecologically, stored carbohydrates have several functions including buffering the asynchrony between resource supply and the demand for growth and supporting regrowth after winter dormancy, fire, herbivory, drought, or frost (Bloom et al., 1985; Slewinski, 2012; Wang et al., 2017). This involves a trade-off between loss in competitive advantage from short-term reserve accumulation and long-term persistence in variable environments (Bloom et al., 1985).

**Figure 1:**
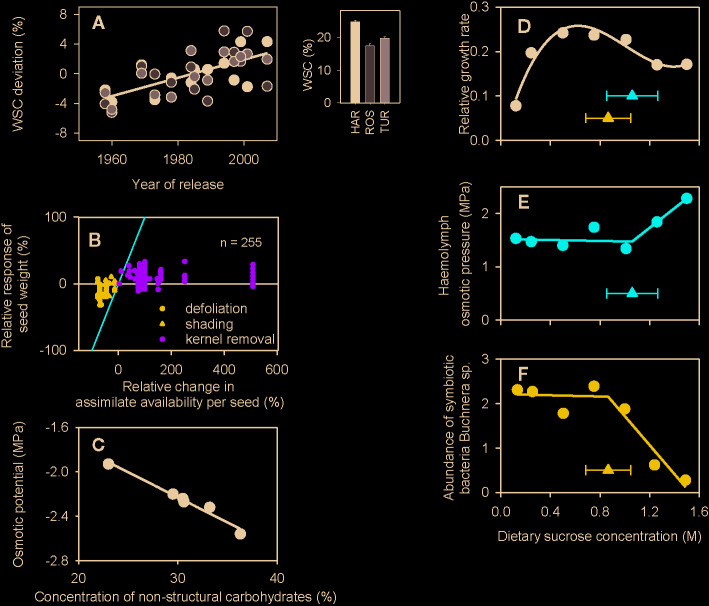
**(A)** Selection for yield over five decades steadily increased the concentration of water soluble carbohydrates (WSC) in wheat adapted to winter-rainfall environments of Australia. Inset is the average across varieties in three environments of South Australia (HAR: Hart, ROS: Roseworthy, TUR: Turretfield) and the scatterplot is the deviation of each variety relative to the environmental mean. The fitted line is the least-square regression (r = 0.66, P < 0.0001). **(B)** Relationship between relative change in wheat seed weight and the relative change in potential assimilate availability per seed produced during seed filling in a number of experiments where source:sink ratio was reduced with defoliation or shading or increased with partial removal of kernels. The lines are the theoretical limits for full source limitation (y = x, red) and full source limitation (y = 0, black). **(C)** Correlation between the osmotic potential and concentration of nonstructural sugars (fructan + sucrose) of wheat plants in a factorial experiment comparing three cultivars (Froid, Brawny, PI 372129) infested with Russian wheat aphid (*Diuraphis noxia*) and uninfested controls. The fitted line is the least-square regression (r = −0.98, P < 0.001). **(D)** Relative growth rate of the pea aphid *Acyrthosiphon pisum* as a function of sucrose concentration in artificial diet. Growth rate is log_e_(day-8 mass/day-6 mass)/2], with each aphid weighed on day 6 and day 8 to the nearest µg. **(E)** Osmotic pressure of the hemolymph of 8-day-old aphids reared on diets with varying concentration of sucrose. **(F)**. Abundance of symbiotic bacteria *Buchnera* spp. in 8-d-old aphids on diets of varying concentration of sucrose. Abundance is 10^−6^ × the number of copies of Buchnera *dnak* gene per ng total DNA. The fitted curves are **(D)** cubic polynomial, **(E, F)** piecewise models with triangles showing the breakpoint ± standard error. Figures have been drawn using data from the following sources: **(A)** Sadras and Lawson (2011); **(B)** Borrás et al. (2004); **(C)** Storlie et al. (1993); **(D–F)** Douglas et al. (2006). The figures have been reproduced with permission by CSIRO Publishing **(A)**, Elsevier **(B)**, Springer Nature **(C)** and Company of Biologists, Society for Experimental Biology (Great Britain) **(D**–**F)**.

Agronomically, stored carbohydrates in cereals and other annuals buffer grain growth especially when stress impairs photosynthesis after flowering (Bidinger et al., 1977; Jones and Simmons, 1983; Hall et al., 1989; Ruuska et al., 2006; Saint Pierre et al., 2010; Slewinski, 2012). Consistent with this role, selection for yield has indirectly enhanced the concentration of nonstructural carbohydrates in the shoot of wheat in dry environments of Australia (**Figure 1A**). Also consistent with this role, wheat grain size is relatively stable in response to severe reductions in source:sink ratio with defoliation or shading (**Figure 1B**). However, grain weight is equally stable in response to large increase in source:sink ratio caused by partial kernel removal (**Figure 1B**), leading to the conclusion that the yield of wheat is dominantly sink-limited during seed filling (Borrás et al., 2004).

Despite the proven role of nonstructural carbohydrates to buffer grain growth, the association between yield and carbohydrate reserves is far from clear (Dreccer et al., 2009; Dreccer et al., 2013; del Pozo et al., 2016; Hoogmoed and Sadras, 2016; Ovenden et al., 2017; Sadras et al., 2019). In the extensive and agronomically robust wheat studies comprising 319 breeding lines and 46 varieties over wide environmental conditions in Australia (Ovenden et al., 2017) and 384 cultivars and advanced semi-dwarf lines in rainfed and irrigated environments in Chile (del Pozo et al., 2016), the correlation between whole-plot harvested yield and concentration of water soluble carbohydrates in the shoot at flowering was low and inconsistent, or even negative. In a set of 16 oat varieties grown in four environments, grain yield correlated negatively with concentration of water soluble carbohydrates in the shoot at flowering (Sadras et al., 2019).

Theory and empirical evidence for a myriad of trade-offs between nonstructural carbohydrates and other fundamental traits explain the weak, inconsistent or even negative correlations with yield. According to the current theory of cereal yield formation, these trade-offs arise in a species-specific critical period when ear survival, floret survival within ears, determination of potential grain size, and root growth overlap with active accumulation of nonstructural carbohydrates (Sadras and Denison, 2009; Slafer et al., 2014). During this period yield is mainly source limited, and empirical evidence supports the antagonistic relationship between accumulation of nonstructural carbohydrates with tillering, ear, and grain number per m^2^ (Lopes and Reynolds, 2010; Dreccer et al., 2013; del Pozo et al., 2016; Sadras et al., 2017; Okamura et al., 2018) and root biomass (Lopes and Reynolds, 2010). For example, the low yield of rice variety Momiroman (608 g m^−2^) in comparison to Teqig (897 g m^−2^) was partially related to a large amount of nonstructural carbohydrates remaining in the crop at maturity (115 g m^−2^ in Momiroman, 24 g m^−2^ in Teqig) (Okamura et al., 2018). There is also a trade-off between remobilization of stored carbohydrates supporting grain fill and the retention of carbohydrates in stem that contributes to lodging resistance (Kashiwagi and Ishimaru, 2004; Kashiwagi et al., 2006; Kashiwagi et al., 2016; Zhang et al., 2017). Nonstructural carbohydrates therefore buffer grain weight but do not contribute consistently to yield and involve significant and widespread trade-offs compromising root and shoot growth and reproduction. Hence the question: does selection for agronomic adaptation unintentionally increase concentration of nonstructural carbohydrates because there are hidden advantages of this trait?

## High Concentration of Nonstructural Carbohydrates Challenges the Osmotic Homeostasis of Aphids

The role of osmotic stress on plant-herbivore interactions has received attention in entomology (Downing, 1978; Fisher et al., 1984; Dixon, 1988; Storlie et al., 1993; Rhodes et al., 1997; Douglas et al., 2006; Alkhedir et al., 2013; Jiang et al., 2016). However, the ecological role of nonstructural carbohydrates mediated by their osmotic effect (**Figure 1C**) has been largely ignored in crop science. Aphids (Hemiptera, Aphidoidea) are the most important insect pests in temperate agriculture (Minks and Harrewijn, 1987), and cereal breeding programs are particularly concerned with aphids as vectors of plant viruses (Trebicki et al., 2017). It is plausible that plant breeders would discard genotypes that favor build-up of aphid populations and feature symptoms of viral diseases (Richards, 1997).

Primarily associated with the concentration of nonstructural carbohydrates (**Figure 1C**), the osmotic potential of the host plant’s phloem sap could be greater than the osmotic pressure of the aphid hemolymph, thus challenging the water balance of the insect. In an early study with *Myzus persicae* grown on sea aster (*Aster tripolium)*, Downing (1978) found that the osmotic pressure of the excreted honeydew was comparable to that of the hemolymph, thus demonstrating the aphid’s ability to reduce the osmotic pressure of the ingested sap. Fisher et al. (1984) later verified that *M. persicae* maintained similar osmolality in hemolymph and excreted honeydew with a 2.2-fold variation in the osmolality of the diet (10 to 30% sucrose w/v). *Sitobion avenae* and *Schizaphis graminum*, but not *Rhopalosiphum padi*, reduced the concentration of total soluble carbohydrates in the phloem sap of wheat after feeding for 72 h (Liu et al., 2020). Polymerization of dietary sugars to oligosaccharides, chiefly the trisaccharide melezitose, is a widespread osmoregulatory mechanism in aphids (Fisher et al., 1984; Rhodes et al., 1997). Further, a filter chamber involved in osmoregulation has been considered adaptive for aphids feeding regularly on a diet of high osmotic potential (Rhodes et al., 1997).

The mean population size of the aphid *S. avenae* declined with increasing concentration of water soluble carbohydrates in cocksfoot (*Dactylis glomerata*), and this effect was attributed to osmotic stress on the aphid (Alkhedir et al., 2013). Conversely, mediated by an increase in leaf turgor, higher osmotic potential of cotton leaves favored *Aphis gossypii* body weight, fecundity, and population abundance, albeit slightly (Jiang et al., 2016). These contrasting responses of aphids to nonstructural carbohydrates may suggest nonlinear relationships between insect fitness and host-plant sugar concentration and osmotic potential. However, in both studies osmotic and C:N ratio were confounded; the influence of plant C:N ratio on insect survival and fitness is well established (Mattson, 1980). Douglas et al. (2006) provide robust, direct evidence of nonlinearity in the response of aphids to diet’s sugar concentration. The relative growth rate of 6- to 8-day-old final instar larvae of pea aphid (*Acyrthosiphon pisum)* varied nonlinearly with sucrose concentration in artificial diets (**Figure 1D**). The relative growth rate was impaired by reduced feeding reflecting the importance of sucrose as a phagostimulant at low dietary sucrose concentrations and by osmoregulation failure at high concentrations. Up to a threshold of 1.06 ± 0.21 M sucrose in the diet, the osmotic pressure of the aphid’s hemolymph was maintained, but above this threshold the breakdown of osmoregulation was apparent (**Figure 1E**). Furthermore, the abundance of symbiotic bacteria *Buchnera* spp., critical in providing essential amino acids to the aphid, collapsed after a threshold of 0.87 ± 0.18 M sucrose in the diet (**Figure 1E**). Both the threshold for osmoregulation of aphid hemolymph and the threshold for survival of symbiotic bacteria were close to the onset of declining relative growth rate (**Figure 1D**) providing a putative causal link between osmotic stress and insect fitness. With concentration of nonstructural carbohydrates over 30% not uncommon in cereals (Storlie et al., 1993; Ovenden et al., 2017; Sadras et al., 2019), the osmotic potential of the plant (**Figure 1C**) could be stressful for aphids (**Figures 1D–F**) (see also Fisher et al., 1984).

## Is It Possible That Selection for Resistance to Aphids Might Unintentionally Favor High Concentration of Nonstructural Carbohydrates in Cereals?

Based on the lack of clear association between nonstructural carbohydrates and yield—the primary breeding target—and the effect of nonstructural carbohydrates on fitness of aphids, we speculate that selection for resistance to aphids might unintentionally favor high concentration of nonstructural carbohydrates, even at the expense of other agronomic traits. In the light of this proposition, the amount and concentration of nonstructural carbohydrates should be regarded as functionally different traits, with amount relevant to the carbon economy of the crop, and concentration playing an osmotic role. The hypothesis that nonstructural carbohydrates in cereals can affect the fitness of aphids *via* osmotic stress can be tested experimentally. Factorial experiments combining cereal phenotypes and aphid species and clones can be designed against a putative curve of insect fitness and behavior against host-plant osmotic potential (cf. **Figure 1D**). Cereal phenotypes with contrasting concentration of nonstructural carbohydrates can be generated with genetic or environmental sources of variation, or both. Assays to quantify insect fitness (Bohidar et al., 1986) and feeding behavior (Givovich and Niemeyer, 1995) in response to specific cereal phenotypes can be combined. To rigorously test our hypothesis, we need to further: (a) test the degree of correlation between concentration of nonstructural carbohydrates in the whole plant, as usually assessed in crop-focused research, and the concentration of nonstructural carbohydrates in phloem as perceived by aphids, and (b) untangle the potential confounded effects of osmotic potential and C:N ratio of plant tissues; the role of N-based osmolytes also deserves attention.

## Author Contributions

VS developed the concept and wrote the manuscript. EF, LB, EG, AM, JA, and AF contributed to discussions and writing the manuscript.

## Funding

VS thanks OECD “Co-operative Research Programme: Biological Resource Management for Sustainable Agricultural Systems” Fellowship to support his work at CSIC in Madrid.

## Conflict of Interest

The authors declare that the research was conducted in the absence of any commercial or financial relationships that could be construed as a potential conflict of interest.
